# Heterocyclic Aromatic Amines in Meat: Formation, Isolation, Risk Assessment, and Inhibitory Effect of Plant Extracts

**DOI:** 10.3390/foods10071466

**Published:** 2021-06-24

**Authors:** Hafiz Rehan Nadeem, Saeed Akhtar, Tariq Ismail, Piero Sestili, Jose Manuel Lorenzo, Muhammad Modassar Ali Nawaz Ranjha, Leonie Jooste, Christophe Hano, Rana Muhammad Aadil

**Affiliations:** 1 Institute of Food Science and Nutrition, Bahauddin Zakariya University, Multan 60800, Pakistan; hrnfoodscientist@gmail.com (H.R.N.); ammarbintariq@yahoo.com (T.I.); 2 Department of Biomolecular Sciences, University of Urbino Carlo Bo, 61029 Urbino, PU, Italy; piero.sestili@uniurb.it; 3 Centro Tecnológico de la Carne de Galicia, Rúa Galicia Nº 4, Parque Tecnológico de Galicia, San Cibrao das Viñas, 32900 Ourense, Spain; 4 Área de Tecnología de los Alimentos, Facultad de Ciencias de Ourense, Universidad de Vigo, 32004 Ourense, Spain; 5 Institute of Food Science and Nutrition, University of Sargodha, Sargodha 40100, Pakistan; 6 Environmental Health Sciences, Faculty of Communication, Arts and Sciences, Canadian University Dubai, Dubai 117781, United Arab Emirates; leonie.jooste@cud.ac.ae; 7 Laboratoire de Biologie des Ligneux et des Grandes Cultures (LBLGC), INRA USC1328 Université ď Orléans, CEDEX 2, 45067 Orléans, France; hano@univ-orleans.fr; 8 National Institute of Food Science and Technology, University of Agriculture, Faisalabad 38000, Pakistan

**Keywords:** heterocyclic aromatic amines, carcinogens, cooked meat, inhibition, natural antioxidants

## Abstract

Heterocyclic aromatic amines (HAAs) are potent carcinogenic compounds induced by the Maillard reaction in well-done cooked meats. Free amino acids, protein, creatinine, reducing sugars and nucleosides are major precursors involved in the production of polar and non-polar HAAs. The variety and yield of HAAs are linked with various factors such as meat type, heating time and temperature, cooking method and equipment, fresh meat storage time, raw material and additives, precursor’s presence, water activity, and pH level. For the isolation and identification of HAAs, advanced chromatography and spectroscopy techniques have been employed. These potent mutagens are the etiology of several types of human cancers at the ng/g level and are 100- to 2000-fold stronger than that of aflatoxins and benzopyrene, respectively. This review summarizes previous studies on the formation and types of potent mutagenic and/or carcinogenic HAAs in cooked meats. Furthermore, occurrence, risk assessment, and factors affecting HAA formation are discussed in detail. Additionally, sample extraction procedure and quantification techniques to determine these compounds are analyzed and described. Finally, an overview is presented on the promising strategy to mitigate the risk of HAAs by natural compounds and the effect of plant extracts containing antioxidants to reduce or inhibit the formation of these carcinogenic substances in cooked meats.

## 1. Introduction

Our diet contains meat as a significant food that contributes valuable constituents and nutrients such as all essential amino acids, high-quality proteins, vitamin B6, niacin, iron, vitamin B12, phosphorus, zinc, and selenium, as well as other biologically active materials, including carnosine, taurine, carnitine, glutathione, creatine, and ubiquinone, that are considered beneficial for human health [1,2,3,4]. Meat is usually cooked to enhance its palatability and safety [5]. Nevertheless, the temperature required for heat processing enables reactions between the components characterized in fish and meat, producing genotoxic compounds [6]. Numerous epidemiological analyses have revealed that a high consumption of meat with different practices of cooking is related to an augmented menace of various cancers in humans, including breast, colon, pancreatic, and prostate cancers [7,8]. Numerous studies have discovered that different procedures of cooking are employed, with elevated temperatures above 150 °C, to cook meat-based products [9], including grilling [10], smoking [11], roasting [12], frying, and barbecuing [13]. As a result of cooking at high temperatures, heterocyclic amines of aromatic compounds are formed.

Heterocyclic aromatic amines (HAAs) can be defined as a main class of compounds that are potentially carcinogenic and extracted from proteinaceous foods during flavor forming and processing using heat [14]. Professor Sugimura was the first person who discovered HAAs, and, at present, 30 different mutagenic HAAs have been identified in protein-rich cooked foods [15]. When specific foods rich in protein, such as pork, chicken, fish, and beef, are cooked for a longer time, a kind of heterocyclic amine of aromatic components is neo-formed [16]. Polycyclic aromatic hydrocarbons (PHAs) and heterocyclic amines (HAAs) are recognized by means of being carcinogenic and/or mutagenic compounds that are available in parts per billion levels of broiled meat [9,17]. 

Nevertheless, numerous epidemiological studies have demonstrated the relationship between the consumption of meat and an elevated risk of occurrence of different types of cancers [18] due to the formation of HAAs, hypothesized to be a cancer menace biomarker [19]. Subsequently, a higher intake of processed and red meat predominantly transports the menace of colorectal tumors [20]. Various analyses have uncovered that meat consumption elevates the risk of numerous kinds of cancer by up to 50% [21]. The International Agency for Research on Cancer (IARC) has reported the classification of processed and red meat and its consumption as a predominant cancer-causing factor in humans. Groups 2A and 1B are two major groups formed with the recommendation of HAA exposure [22].

Studies have demonstrated that regular intake of grilled meat with HAAs may enhance the risk of cancer in different organs of humans [21]. It has been recommended that the exposure of these components to humans should be lessened [23]. Effective HAA inhibitors have been identified, which are needed significantly. Moreover, antioxidants that are added to meat before cooking are considered the most effective and promising HAA inhibitors at different levels [24]. It is suggested that the Maillard and free radical reactions have a combined effect on the production of HAAs, establishing pathways. In the initial levels of the Maillard reaction, antioxidants may responsibly act as scavengers of free radicals, which decreases the formation of HAAs [25]. 

Products from natural sources have been used for centuries [26,27]. Plants, because of their phytochemical profile, have emerged as the best resource to fight against different disorders and diseases [28,29]. Numerous systems of food have been studied with the aim of reducing or inhibiting HAA formation, with the commitment to decrease the developing risk of chronic diseases. Different methods of cooking, utilizing food additives with the presence of natural antioxidative compounds such as phenolics [30], can cause a reduction in HAA formation in cooked meats. The presence of natural antioxidants in herbs and spices has received considerable attention for use as proficient inhibitors, also working against the mutagenic formation of HAAs in processed meat [31,32,33]. Pomegranate seed extracts [34,35], grape seed extracts [36] olive and apple extracts [28,37], spices such as lemongrass, turmeric, and torch ginger [32,38], herbs such as savory, thyme, and oregano [39], galangal and fingerroot [15], vitamin E that is lipid-soluble [40], and vitamins that are water-soluble [41] have been reported to inhibit HAA formation in the food systems.

The existence of HAAs in sufficiently heated food matrices and the monitoring of the negative effects of these toxic compounds on human health and the inhibition of their existence in cooked food is an obligatory requirement. This review demonstrates the delivery of detailed information about HAAs that is based on the knowledge from proceedings to advanced studies. Numerous pretreatments and methods are applied in different samples of food and clarify the different techniques for the analysis, determination, and isolation of HAAs. Finally, an overview is presented on a promising strategy to mitigate the risk of HAAs by natural compounds and the effect of plant extracts containing antioxidants to reduce or inhibit the formation of these carcinogenic substances in cooked meats.

## 2. Occurrence of HAAs

The identification of HAAs in various foods, such as cooked fish and meats, products of meat, extracts of meat [42], coffee [43], extracts of meat oils [44], food flavors, and alcoholic drinks, and in environmental sources (surface and air–water) and biological and industrial fluids as well as cigarette smoke and kitchen fumes have been determined [45].

Quantities of HAAs vary in the case of meat from different animal species, for example, fish, poultry, pork, and beef. For example, a great occurrence of PhIP (2-amino-1-methyl-6-phenylimidazo[4,5-b]pyridine) and IFP (2-amino-1,6-dimethyl-furo[3,2-e]imidazo[4,5-b]pyridine) in poultry has been observed while more MeIQx (2-amino-3,8-dimethylimidazo[4,5-f]quinoxaline) is observed in pork, fish, and beef [46]. It is well observed that MeIQx, after PhIP, is regarded as the second most abundant HAA [8]. The household methods of cooking mostly cause the formation of HAA levels in meat in small ranges (0.1–50 ng/g) [47].

Gibis and Weiss [48] conducted a study to assess the impact of precursors creatine, creatinine, and glucose on the formation of heterocyclic aromatic amines in grilled patties of various animal species. In their study, the meats of veal, beef, pork, lamb, horse, venison, turkey, chicken, and ostrich were investigated. They found that the MeIQx content in grilled patties of various animal species ranged from 0.49 to 1.35 ng/g, 4,8-DiMeIQx ranged from 0.05 to 1.27 ng/g, PhIP ranged from 0.17 to 10.47 ng/g, Norharman ranged from 0.55 to 1.88 ng/g, and Harman ranged from 0.07 to 3.18 ng/g [48]. Similarly, Gibis and Loeffler [49] conducted a study of creatine and glucose on the formation of heterocyclic amines in grilled chicken breasts. They found different HAA ranges, namely, 0.55–2.16 (Harman), 0.74–2.29 (Norharman), 1.49–9.05 (PhIP), and 0–1.12 (MeIQx) ng/g [49]. The significant occurrence of PhIP and IFP in poultry was observed, while more MeIQx was observed in pork, fish, and beef [46].

## 3. HAA Formation

The amines of heterocyclic compounds are divided into groups of carcinogenic and mutagenic compounds that are produced on the surface of foods rich in protein, such as fish and animal meats [38], and result in the development of the Maillard complex reaction during roasting and frying [50]. Both polar and non-polar HAAs (Harman and Norharman) are produced at lesser temperatures [18]. It is suggested that free radical compounds play a significant role in the IQx- (2-amino-3-methyl-3 H-imidazo [4,5-f]quinoxaline) and IQ- (2-amino-3-methyl-3 H-imidazo[4,5-f)quinolone) type HAA formation that is mostly developed through Strecker degradation radicals and creatinine condensation (pyrazines and pyridines) reactions. The Maillard reaction is mostly applicable during amino acid reactions and glucose generation [51].

**Figure 1:**
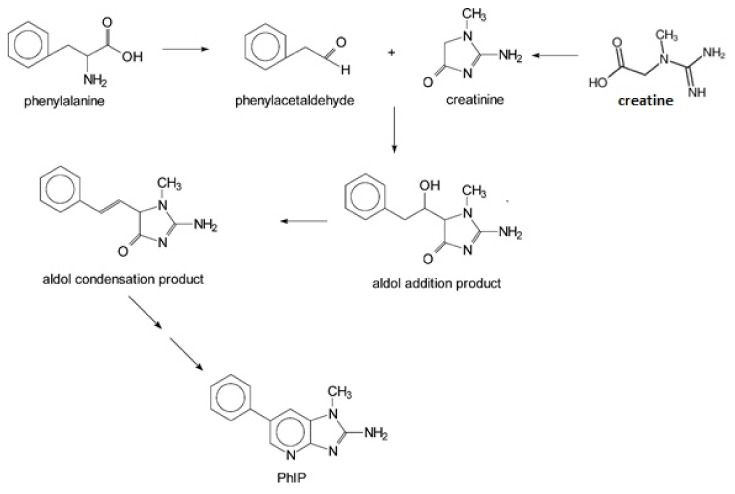
PhIP chemical formation reaction.

Reducing sugars, creatinine, and phenylalanine can be distinguished in the formation of PhIP. In these studies, scientists have identified the reaction, which leads to the formation of PhIP, by following steps through a model experiment. Firstly, the formation of phenylacetaldehyde occurs from phenylalanine through the degradation of the Strecker reaction. Then, the formation of phenylacetaldehyde is complete during a reaction with aldol and creatinine to develop an additional aldol product. PhIP is formed from the condensation of aldol by a subsequent condensation reaction [52]. Figure 1 shows the reaction mechanism of compound formation.

### Precursors and Factors Affecting HAA Formation

Reducing sugars, such as fructose and glucose, are produced directly by sucrose hydrolysis of free amino acids and creatinine, available naturally in muscular mammalian tissue and referred to as the potential precursors, accountable for HAA formation. Moreover, various precursors such as creatinine, free amino acids, nitrogenous bases, sugars, protein, and nucleosides are produced at different levels of raw material processing [53]. The variety and content of HAAs in cooked foods are determined by various factors such as the quantity of meat, fresh meat storage time, the kind of raw material, physical aspects of the heat conduction such as duration of heating and temperature, equipment for cooking, the method of processing, the presence of inhibitors or enhancers, the level of doneness, the activity of water, and, particularly, pH. All the abovementioned factors speed up the production of HAAs and have a greater effect on the reaction of HAA formation [18,54].

HAAs are not affected by the variations in pH in the actual sense. HAA precursors are sensitive to pH changes by which the formation of HAAs can ultimately be affected. Enhanced pH levels can modify the formation pathways of HAA precursors and lead to minimum HAA production. HAAs are sensitive to the contents and oxidation of lipids, and their formation is also affected by the availability of antioxidants [55].

Polak et al. [56] conducted research on the influence of temperature on the formation of heterocyclic aromatic amines in pork steaks. They found that HAA levels increased with increasing grilling temperature. A very similar study was conducted by Knize et al. [57] to assess the effect of cooking time and temperature on the heterocyclic amine content of fried beef patties. They also reported the elevation of HAA levels with increased temperature and time.

In addition, the quantity of meat fat has a noticeable impact on the formation of HAAs, and it is believed that HAA concentration is lower in fat-rich meat than other kinds of meat. The transportation of high heat is required in fat-rich meat to enhance the time of cooking compared to lean meat [58]. The influence of animal species on the formation of HAAs in marinated meat has been described [16].

It is hypothesized that the higher concentrations of free amino acids and total creatine are linked to the development of HAAs in more quantity during the treatment of thermal heat; a correlation that was quite strong was noted between these features. Despite the detailed fact that the concentration of HAAs somehow originates from reducing sugars, only frail interdependence was attained from these parameters [59]. It was revealed that numerous HAAs could be obtained by amino acids heated with creatine with no addition of sugar to the system of the model. Amino acids play a more substantial role in HAA formation than sugar. Creatine is considered as a major precursor in the formation of polar HAAs, which carry a dominant reaction with products obtained by Maillard reaction to form more polar HAAs [60]. Bordas et al. [61] revealed that the quantities of Harman, Norharman, and MeIQx were enhanced in a meat-flavored model system with a 50- fold increase in glucose. Tai et al. [62] observed that the quantities of HAAs were enhanced in fried fish, especially when frying by adding 9% sugar, although reduced when 19% sugar was used when frying. The results predict that the addition of more sugar during frying attributes to the production of Maillard products with less HAAs. The creatinine that reacts with Maillard products also acts as a precursor for HAA formation [18]. Wu et al. [63] described the process of cooking in ethanol and demonstrated that ethanol enhanced IQ and IQx formation, depending on the dose concentration. Increased concentrations of ethanol also increased the rate of browning in the solution [63]. The contents of Norharman and Harman were increased and significantly affected by the presence of fructooligosaccharides, which was observed predominately in the previous study [64]. Liao et al. [65] observed the reactions of Ile, Glu, Phe, Tyr, Ser, and Thr with glucose, which developed HAAs in pork floss.

Skog [66] observed that Tyr boosted the production of IQx compounds, and high quantities of Ala, Phe, and Gly favored PhIP and MeIQx formation in moist states. It has also been reported that soy albumin proteins, globulin proteins, gluten, and casein are formed with HAAs at temperatures above 250 °C; nevertheless, the process has not been completely clarified [67].

## 4. Types of HAAs

HAAs are considered analytes that are hazardous, and, therefore, they are categorized into two major groups according to their reactions of formation. They can be grouped into pyrolytic and thermic HAAs. Thermic HAAs can be produced by free amino acid reactions containing hexoses and creatinine at temperature ranges between 100–300 °C. They are also recognized as aminoimidazoazarenes and the IQ type. Pyrolytic HAAs are produced at temperatures above 300 °C, and pyrolytic reactions are applied between proteins and amino acids for the formation of hazardous compounds [18,68].

In another survey, HAA formation was detected diversely. The study described that HAAs are divided into two major groups known as polar HAAs (IQ type) and non-polar HAAs (non-IQ type), depending on their structure, temperature, and formation pathways [69]. The group of polar compounds is formed from amino acids, creatinine, and carbohydrates at a temperature range between 150–250 °C. It is observed that polar HAA compounds initiate their formation with the Millard reaction, especially various free amino acids and glucose between reducing sugars [47,70]. The creatinine condensation terminates HAA formation with pyrazine and pyridine and with the intermediate free radical of the Maillard reaction. The non-polar HAA group is formed from the pyridoindole or dipyridoimidazole moiety from amino acid pyrolysis, such as glutamic acid, lysine, phenylalanine, tryptophan, and ornithine, except for creatinine, at temperatures more than 250 °C [41,71].

In another study by Sahar et al. [72], HAAs are classified based on ring structure, as follows:

- Five-membered amines of a heterocyclic nature;
- Six-membered amines of a heterocyclic nature.

Five-membered amines of heterocyclic compounds include five ring atoms. The structure of these cyclic compounds is comprised of four carbon atoms and one nitrogen atom. The main examples of these compounds are pyrrole and nicotine. The six-membered structure of amines of heterocyclic compounds show more similarity towards benzene; the difference is where nitrogen substitutes the carbon atom in the structural ring [72].

Abbreviations, names of compounds, structure, molecular mass, chemical formulas, and the non-polar and polar presence of HAAs in different varieties of foods are mentioned in Table 1.

## 5. Quantification and Identification

Progressive extraction procedures and instrumental methods have been applied for the detection and separation of HAAs. According to the kind of preparation of the sample, the experiment’s sensitivity, and the choice of systematic equipment, laboratory, and accessible facilities will be dissimilar [14].

Colorimetric procedures have been established for the measurement of amines, including biogenic compounds and copper ions. This experiment includes a detection limit of about 5 mg/kg and enumerates the amines of biogenic compounds by 496 nm value of absorbance with the assistance of a spectrophotometer. The procedure has the benefit of considering only 45 min of analysis. Furthermore, the method is also determined as a method of cost-effectiveness and does not need specialized laboratory equipment [95].

Fluorescence synchronous spectroscopy has been utilized to enumerate the quantity of numerous HAAs in meat samples that were heated by the measured condition [72]. The most frequently utilized procedure of sample treatment for the detection of HAAs is the adapted Grüter and Gross method, which depends on the SPE’s three steps of purification and alkalization, for example, the columns coupled with diatomaceous earth, RP-phase 18, and sulfonic propyl acid [96]. Such a multistep procedure of pretreatment takes substantial time, with a large number of reagents and chemicals. Briefly, the recoveries attained from these frequently used approaches to pretreatment are unacceptable for all HAAs targeted. In the Grüter and Gross method, the efficiencies of extraction through all levels of purification procedures are better than 53% α-carboline amines and 41% PhIP. Similarly, utilizing various procedures, the authors of [97] showed an instantaneous investigation of 14 HAAs in barbecued samples of salmon and sardines. The establishment of electrophoresis of the capillary process and the procedure of in-line preconcentration for the determination of 16 HAAs simultaneously were examined [98].

Yan et al. [99] anticipated an accurate and simple acetonitrile extraction method of pretreatment, equipped with high ultra-performance liquid chromatography to mass tandem spectrometry for the instantaneous measurement of 17 HAAs in meat products. In addition, recent studies have measured instantaneous 15 HAAs in grilled fish utilizing high ultra-performance liquid chromatography; the solid extraction phase (SPE) with ionization electrospray mass tandem spectrometry (ES–UHPLC-MS) has been described by Feng et al. [100].

In recent studies, nine retrievals for HAAs were more than 50% extra (51–68%), although 1-mehyl-3-amino 5H-indole pyrido, 3-amino 1,4-5Hdimethyl- indole pyrido, PhIP, 2-amino-9H-pyrido-indole, and (MeAαC) 2-methyl-amino-3-9H-indole-pyrido had the range of 23%–32% (Costa et al., 2009). In additional recent studies, 15 targeted recoveries of HAAs were ranged between 35%-70%, commonly consumed in pan-fried beefsteak. Among the 15 HAAs targeted, the rates of recovery for 4,8-trimethyl-2-amino-3 quinoxaline imidazo (4,8-DiMeIQx) and (Norharman) 9H-pyrido-indole reached 42.7% and 35.9% correspondingly [101]. Subsequently, the problems of analysis for a long time preparation had a recovery that was unsatisfactory during the process of pretreatment, which designates the requirement for an improved analysis procedure to enumerate various HAAs instantaneously. Currently, HPLC is only the technique that can moderately, accurately, and reliably quantify and detect HAAs in meat samples of cooking. Consequently, it is the utmost reliable technique for the analysis of food. Presently, the extraction of HAAs is determined by advanced methods such as the extraction phase of solids and the extraction of liquids and solids. Ultra-performance liquid chromatography (UPLC), gas chromatography (GC), and mass spectrometry (MS) specifically analyze the phases [102]. Sahar et al. [72] utilized fluorescence synchronous spectroscopy (FSS) coupled with HAA quantification of chemometrics in samples of grilled meat. Jautz et al. [103] quantified HAAs in fried meat by HPTLC/UV-FLD and HPLC/UV-FLD. Sentellas et al. [98] determined HAAs by capillary electrophoresis coupled to mass spectrometry using in-line preconcentration. Vanderlaan et al. [104] used enzyme-linked immunosorbent assays for HAA determination.

## 6. HAA Risk Assessment

The salmonella or Ames test demonstrates that maximum HAAs are extremely mutagenic [105], and HAAs are entirely recognized as carcinogenic compounds in toxicology studies [14]. Cancer development is mostly lead by HAAs, which cause the mutation of genes and abnormal patterns of growing cells [106]. Several research discoveries have established that HAAs can modify DNA, such as chain hydrogen bond breaking in DNA, mutations in site, and deletions and insertions in DNA [9]. These compounds show extreme mutagenesis; they have almost ten times the carcinogenicity of other poisonous compounds such as aflatoxins B1, nitrosamines, and pyrene benzo compounds [107]. The HAAs are more than 100-fold more mutagenic than aflatoxins B1 and 2000-fold more mutagenic than pyrene benzo compounds when compared to other mutagens in food [81]. The HAA compounds are engrossed in the small intestine of the body and, afterward, move to the liver and are triggered in this organ [108]. The activation of metabolic HAAs begins with N-oxidation by enzymes of the cytochrome, and the reaction of acetylation is compulsory for this stage of activation [109]. The product of N-acetoxyamine, which is mentioned above in the reaction, is a reactive molecule that can bring damage to DNA and cause mutations [110].

According to the inspection description of the National US Program of Toxicology, HAA uptake ranges above 17 ng/kg of body weight show a strong correlation with the intake of meat-based cooked products. The continuing survey also commented that individuals who consume products of meat are mostly accountable for HAA accumulation in the body [111]. The exposure of HAAs to the human body is also affected by the method of cooking as well as the kind of food. Many other factors, such as frequency of consumption and size of portion, are also involved [112]. The indication of daily estimated levels of HAA consumption ranges from 0–15 mg/day according to the diet per person. In the current study, HAA consumption is less than 1 mg, most probably in the case of consumption of 100 g of medium chops in the control group, with the molecular weight of chitosan heated at 250 °C, which acquired the highest quantity of HAAs (4.21 ng/g) [54].

The Europe Council advises that the quantity of HAA consumption must have a lower quantity than 1 µg per day [113]. Although the quantity of daily consumption of HAAs is diverse in numerous epidemiological surveys, the studies have assessed that the calculated daily consumption of HAAs has a quantity of about 420 ng/d per person. Wolk [114] emphasized that 50 g per day consumption of meat increases the carcinogenic probability; the colonic cancer rate is estimated at 18%, prostate about 4%, breast 9%, and pancreatic cancer at 19%. The mutagenic compounds of HAAs noticed in commercial products of meat and heated meat is based on homemade standards; the level ranges from 1–100 ng g^−1^ [99]. 

Frequently, cases of colon cancer are reported to be about 7–9% due to red meat consumption with HAAs [115]. Rohrmann et al. [116] investigated that HAA consumption can be above 41.4 ng/day, which increases the colorectal tumor risk. Szterk [117] showed that digestion in the digestive tract of humans may involve HAAs that are unrestricted and demonstrate a significant carcinogenesis role. Consequently, the hazard and formation of HAAs in broiled foods have created public anxiety for the health of consumers. The surveys of epidemiological studies have established that the risk is enhanced in colon, breast, pancreatic, prostate, stomach, and other cancers, which are linked with well-cooked red meat intake, related closely to HAAs [118,119,120,121,122]. Cancer in the lung is mostly caused by HAAs that are responsible for cancer onset [123]. The HAAs’ carcinogenicity has also been recognized in animal experiments. Recent studies have demonstrated the HAAs’ mutagenicity in bacteria [124], investigational animals, and the cells of mammals [125]. Four HAAs (MeIQ, IQ, PhIP, and MeIQx) are recorded in the 11th Carcinogens survey report by the Department of US Human and Health Services in 2005 as constituents that are reasonably expected to cause carcinogenesis in humans.

## 7. HAA Inhibition by Natural Extracts

Various risks are linked with the intake of HAAs; decreasing the exposure of HAA development by diminishing proteinaceous foods, cooking with reduced cooking time and temperatures [55], and a reduction in meat storage are also recommended. Szterk and Jesionkowska [42] suggested the consumption of meat that has more fat rather than low-fat meat. Marinating meat with diverse spices and herbs [39,126] and the pretreatment of meat [53] are the methods proposed to reduce the formation of HAAs in meat. 

Numerous studies have exposed the effects of inhibition with synthetic antioxidants (butylated hydroxytoluene (BHT), butyl tertiary hydroquinone [69], butylated hydroxyanisole (BHA) [127]) or natural antioxidants such as polyphenols [128], white green and black flavonoids from tea [129,130], the chrysin flavonoid [85], flavonoids in citrus [131], ellagic acid [132], vitamin E and C [40], propyl gallate (PG), extracts of plant or fruit [126,133], beer [134], wine [135], and numerous spices [25] on the formation of HAAs in meat-based products. Polyphenols have been designated for the inhibition of HAA production via the free radicals formed from the Maillard reaction [136].

Pomegranate seed extracts [35], olives and apples [37], herbs such as savory, oregano and thyme [39], the seeds of grapes [36], spices such as torch ginger, lemongrass, and turmeric [38], soluble lipid vitamin E [40], vitamins soluble in water [41], and galangal and fingerroot [15] have been suggested to prevent HAA formation in systems of food. The accumulation of black pepper powder and turmeric [55], hibiscus [137], and extracts of marjoram, oregano, rosemary, coriander, and savory [39] are regarded as ingredients of marinades before cooking that decrease HAA content. The formation of HAAs was suppressed by the formation of free radicals scavenging in HAA formation pathways [122].

The reduction in the degree of HAAs seems to be based on the concentration and potency of the plant extracts as well as extractions of HAA concentration in the samples of meat. The results show that except for the determination of the property of antioxidants, which is an important feature in the reduction of HAA formation, other features in the ingredients of marinades have a major contribution to the extraction of precursors from the HAAs at different concentrations, including amino acids and glucose that also influence the reduction or formation of HAAs [33]. The inhibition of total formation of HAAs occurred at different percentages of 47%, 50%, 46%, and 54% by galacto-oligosaccharide, isomalto-oligosaccharide, inulin, and fructo-oligosaccharide applications, respectively, at the dose of 1.5 g/100 g in patties of ground beef [138]. Gibis et al. [139] reported that HAA formation could be inhibited with the addition of fibers of cellulose. In other research, Hasnol et al. (2014) described a reduction in PhIP, MeIQ, Norharman and MeIQx levels in grilled marinated chicken by using honey, table sugar, and brown sugar before cooking [7]. Balogh et al. [73] conducted a study in which they reported that (1–10%) vitamin E addition before frying in the beef can meaningfully decrease the formation of HAAs at various levels. The inhibition of tocopherol is the reason behind the free radical’s formation, which is essential as one of the HAAs’ precursors [73].

Tengilimoglu-Metin et al. [140] described that the extract of hawthorn at 1% and 0.5% reserved the formation of Norharman and Harman correspondingly. It mostly happens due to the occurrence of phenolic complexes comprising proanthocyanins and flavonoids in the extracts of hawthorn [140]. Sabally et al. [115] observed that the extract of polyphenol in the peel of an apple is functional to the patties’ surface, broiled at 225 °C. The results found by Keşkekoǧlu and Üren [35] exposed that the extract of pomegranate seed, added to meatballs of chicken, reduced total HAAs by 49%. Viegas et al. [8] described that marinades comprising white wine, mixed herbs, and beer decreased 4,8-DiMeIQx generation. Fatih Oz [141] described that the addition of red pepper to meatballs of high-fat decreased the 4,8-DiMeIQx level, with value ranges from 1.77–0.54 ng/g. Moreover, Dong, Lee, and Shin [23] described that marinade added with a sauce of 8% extracts of water with lotus and olive leaves to fried chicken breasts reduced MeIQx, PhIP and MeIQ formation by 51%, 79%, and 23%, correspondingly. Shin and Ustunol [142] also detected DiMeIQx, PhIP, and MeIQx reduction levels in fried marinated chicken utilizing lemon juice, buckwheat, or clover honey. Tengilimoglu-Metin and Kizil [17] exposed that the extract of artichoke, added before cooking, may be effective in limiting the formation of HAAs due to the activities of the antioxidant associated with polyphenolic compounds such as flavonoids and acids.

Vitaglione and Fogliano [136] exposed that tomatoes include carotenoids that can decrease MeIQx and IQ levels in samples of meat by 36% and 11%, correspondingly, at the 1000 ppm rate. Furthermore, quercetin flavonoid compounds decreased HAAs by up to 67% at 10 ppm concentration [136]. Rauscher et al. [143] utilized carrot, apricot, tomato, orange, Brussels sprout, peppers, and sprout extracts for the inhibition of HAAs and revealed an antimutagenic capability due to the availability of numerous bioactive components such as carotenoids and xanthophylls. The seeds of corn also comprise anthocyanins that decrease mutagenicity, with PhIP most affected [144]. Similarly, cacao comprises pro-anthocyanides that prevent the effects of carcinogens due to HAAs. The anticancer activity proficiently present in the extract of cherry due to anthocyanins has great potential to decrease the level of HAAs by the action of antioxidative activity [136]. Edenharder et al. [145] reported that grapes, blueberries, spinach, watermelon, parsley, and blackberries decreased HAA concentrations in patties of beef. The inhibitory effect of different plant extracts against various HAAs in meat-based products is presented in Table 2.

## 8. Suggested Mechanisms for an Inhibitory Effect to Decrease Potential Carcinogenic Constituents 

### 8.1. The Action of Antioxidative Products 

Meat products consumed, including processed meat and red meat, may result in the incorporation of possible carcinogenic substances developed by lipid oxidation in the components of meat during cooking. Several studies have confirmed that natural products can cause a reduction in the occurrence of carcinogenic substances developed during meat lipid peroxidation. Natural products that are utilized to reduce the degradation or oxidation of meat components can inhibit the formation of carcinogenic substances after or during cooking, such as the free radicals involved in HAA formation [132]. 

Vitaglione and Fogliano [136] described that antioxidants could prevent mutagen formation through free radical scavenging and quenching. Antioxidants can reduce the premutagens’ biotransformation into reactive species of metabolites by stimulating enzyme detoxification or reactive molecule scavenging activity [136]. Natural products can perform antioxidative activities such as a decrease in oxidative stress or reduction in the oxidation of meat components [161,162].

An antioxidant can protect against the toxicity of iron that chelates ferrous iron and inhibit its reaction with oxygen, peroxides, and chelated iron and trap already developed free radicals. Phenols available in olives rapidly decreased heme-induced peroxidation of lipids and scavenged radicals of (1, 1-diphenyl-2-picrylhydrazyl) DPPH. Vitamin E can inhibit the damage induced by iron, and vitamin C can cause the reduction of free iron into ferrous iron, stimulating the propagation and initiation of free radical reactions [163]. Gobert et al. [164] explained that fruits and vegetables contain polyphenolic extracts that hinder the process of gastric digestion, as revealed through total lipids and contents of heme iron. Consequently, a reduction in heme iron oxidation in products of meat using natural products prevents potential carcinogenic substance formation as meat products are the major source of heme iron [164]. The mechanism of antioxidant activities of natural products is shown in Figure 1. 

### 8.2. Inhibition of Maillard Reaction 

Several inhibitors have been found in the studies of the Maillard reaction in specific foods [165]. The Maillard reaction is responsible for the possible formation of carcinogenic substances in meat products, and Maillard reaction inhibition is required to reduce the potential carcinogenic substances via the utilization of natural products in the products of meat. The type and concentration of HAAs are closely related to meat type, pH, water activity (aw), and the methods of cooking used, such as grilling, barbecuing, frying, roasting, or baking. The transfer of heat to the meat products’ surface and precursors of HAA by mass transport apparent to the meat crust also affect the formation of HAAs [166]. 

**Figure 2:**
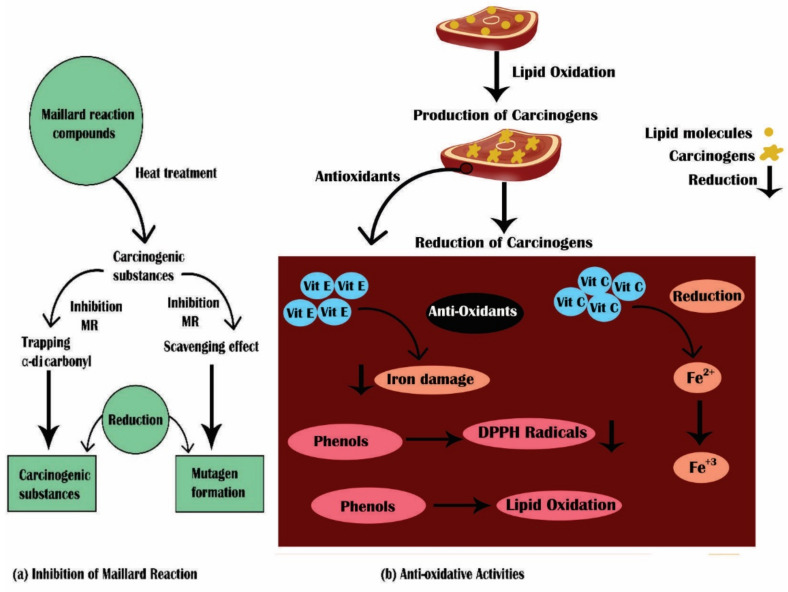
Proposed mechanism of action of natural products on carcinogenic substances. (**a**) Inhibition of the Maillard reaction; (**b**) antioxidative activities.

During treatments of heat, the Maillard reaction can decrease HAA formation. Račkauskienė et al. [167] discussed that beetroot (*Beta vulgaris*) effectively reduced the Maillard reaction’s substance formation in meat and milk protein. The spices’ role, particularly garlic and onion, as HAA formation inhibitors, has been described [168]. Garlic and onion comprise a high quantity of sulfur compounds, such as thiosulfinates and allicin [166]. Schoch [169] suggested that garlic, containing sulfur compounds such as cystine diallyl disulfide, S-oxides S-alkenyl cysteine, and dipropyl disulfide, possesses an inhibitory impact on HAA formation. The inhibitory impacts of different compounds of sulfur, such as acetylcysteine, cysteine, diallyl disulfide, and glutathione, on MeIQx formation have been shown iin a model system of meat [169]. Maillard reactions are initiated by α-dicarbonyls, and polyphenol compounds can hinder Maillard reactions by the trapping of α-dicarbonyls. Consequently, we postulate that the trapping of α–dicarbonyl and the ability to scavenge of flavonoids or phenolic compounds available in natural products can reduce HAA formation from the Maillard reaction in products of meat. Sugar is a significant factor for decreasing mutagenicity. The content of sugar may be imperative for reducing the formation of mutagens due to the reaction of Maillard [170]. Possible mechanism for the inhibition of the formation of potential carcinogenic substances in products of meat by using natural products is described in Figure 2.

## 9. Conclusions

In conclusion, heterocyclic aromatic amines are potential carcinogens that can lead to a number of different cancer types. Cancer risk is associated with a high intake of well-done cooked meats due to the induction of heterocyclic aromatic amines. Hence, it is necessary to inhibit or minimize the formation of these potent mutagenic and/or carcinogenic HAAs. There are a number of possible means to inhibit the heterocyclic aromatic amines, but, among the different mitigation techniques, the utilization of natural plant extracts containing antioxidants has shown promising inhibitory effects against HAA formation. The studies included in Table 2 of the current study critically articulate the heterocyclic amine inhibitory effect of some plants. For that reason, the addition of plant extracts instead of synthetic antioxidants is recommended before intensive cooking of meat-based products. 

## Figures and Tables

**Table 1:** Structure, abbreviation, compound name, chemical formula, molecular mass, and presence of polar and non-polar HAAs in foods.

Structure	Abbreviation/ Compound Name	Chemical Formula/ Molecular Mass	Food	Reference
**Polar HAAs**				
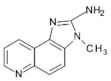	IQ 2-Amino-3-methylimidazo[4,5-f]quinolone	C_11_H_10_N_4_ 198.2	Beef, Goose (Breast and Leg), Suck, Fish (Salmon, Mackerel, Sardine, Whiting, Trout, Sea bass), Chicken (Breast), Duck (Breast), Hamburger Patties, Meatball, White Wine, Red Wine, Soup Cubes	[73,74,75,76,77,78,79,80]
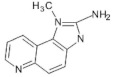	Iso-IQ 2-Amino-1-methylimidazo[4,5-*f*]quinolone	C_11_H_10_N_4_ 198.2	Beef Stock Extract	[81]
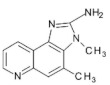	MeIQ 2-Amino-3,4-dimethylimidazo[4,5-ƒ]quinolone	C_12_H_12_N_4_ 212.3	Beef, Goose (Breast and Leg), Suck, Fish (Salmon, Mackerel, Sardine, Whiting, Trout, Sea Bass), Hamburger Patties, Sausages, White Wine,	[11,54,73,75,76,78,79]
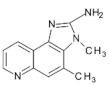	IQx 2-Amino-3-methylimidazo[4,5-ƒ]quinoxaline	C_10_H_9_N_5_ 199.3	Goose (Breast and Leg), Fish (Salmon, Trout, Sea Bass), Chicken Burger, Chicken Nuggets,	[74,76,78]
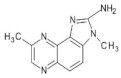	8-MeIQx 2-Amino-3,8-dimethylimidazo[4,5-ƒ]quinoxaline	C_11_H_11_N_5_ 213.3	Beef	[73]
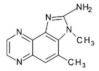	4-MeIQx 2-Amino-3,4-dimethylimidazo[4,5-ƒ]quinoxaline	C_11_H_11_N_5_ 213.3	Beef	[73]
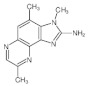	4,8-DiMeIQx 2-Amino-3,4,8-trimethylimidazo[4,5-ƒ]quinoxaline	C_12_H_13_N_5_ 227.3	Beef, Lamb Camel, Beef, Goose (Breast and Leg), Fish (Salmon, Whiting, Sea Bass), Suck, Bacon, Chicken (Breast), Duck (Breast), Meatball, Sausages, Soup Cubes,	[9,11,54,68,75,76,77,80,82,83]
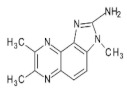	7,8-DiMeIQx 2-Amino-3,7,8-trimethylimidazo[4,5-ƒ]quinoxaline	C_12_H_13_N_5_ 227.3	Beef, Suck, Goose (Breast and Leg), Fish (Salmon, Sardine, Whiting, Trout)	[73,74,75,76]
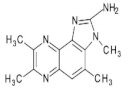	TriMeIQx 2-Amino-3,4,7,8-tetraimethylimidazo[4,5-ƒ]quinoxaline	C_13_H_15_N_5_ 241.3	Grilled Lean Beef	[40]
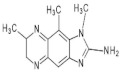	7-MeIgQx 2-Amino-1,7-dimethyl-1*H*-imidazo[4,5-*g*]quinoxaline	C_11_H_11_N_5_ 213.2	Pork (Chops, Bacon, Sausage Patties), Chicken (Breast, Boneless)	[11,84]
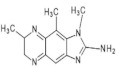	7,9-MeIgQx 2-Amino1,7,9-tridimethyl-1*H*-imidazo[4,5-*g*]quinoxaline	C_12_H_13_N_5_ 227.3	Fried Beef, Barbecued Chicken	[85]
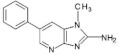	PhIP 2-Amino-1-methyl-6-phenyl-imidazo[4,5-*b*]pyridine	C_13_H_12_N_4_ 224.3	Cheese, Beef, Lamb Camel, Suck, Bacon, Pork Loin, Fish (Salmon, Mackerel, Sardine, Whiting, Trout, Sea Bass), Chicken (Breast), Duck (Breast), Chicken Burger, Meatball, Sausages, Beer, White Wine,	[11,68,73,75,76,77,79,82,86,87,88]
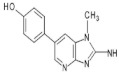	4′OH-PhIP 2-Amino-1-methyl-6-(4′hydroxyphenyl)-imidazo[4,5-*b*]pyridine	C_13_H_12_N_4_O 240.6	Fried and Grilled Chicken Breast	[89]
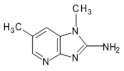	DMIP 2-Amino-1,6-dimethylimidazo[4,5-*b*]pyridine	C_8_H_10_N_4_ 162.2	Pork Fried Meat Emulsion	[90]
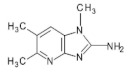	1,5,6 TMIP 2-Amino-1,5,6-trimethylimidazo[4,5-b]pyridine	C_9_H_12_N_4_ 176.2	Grilled Beef, Taiwan Sausages	[11,91]
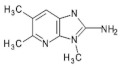	3,5,6 TMIP 2-Amino-3,5,6-trimethylimidazo[4,5-b]pyridine	C_9_H_12_N_4_ 176.2	Commercial Cooked Unknown Meat	[58]
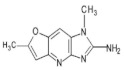	IFP 2-Amino-1,6-dimethyl-furo[3,2-*e*]imidazo[4,5-b]pyridine	C_10_H_10_N_4_O 202.3	Pork (Bacon, Sausage Patties), Chicken (Breast, Boneless)	[11,84]
**Non-polar HAAs**				
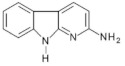	AαC 2-Amino-9*H*-pyrido[2,3-b]indol	C_11_H_9_N_3_ 183.2	Cheese, Goose (Breast and Leg) Suck, Bacon, Fish (Whiting), Meat-based Infant Foods, Coffee	[9,54,71,75,76,83,87,92]
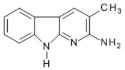	MeAαC 2-Amino-3-methyl-9*H*-pyrido[2,3-b]indol	C_12_H_11_N_3_ 197.2	Cheese, Goose (Breast), Meat-based Infant Foods, White Wine, Red Wine	[54,79,87,92]
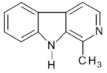	Harman 1-methyl-9*H*-pyrido[4,3-b]indole	C_12_H_10_N_2_ 182.2	Soft Cheese, Beef, Mutton, Lamb Camel, Bacon, Chicken, Meatball, Sausages, Meat-based Infant Foods, Coffee	[11,16,65,76,86,88,92,93]
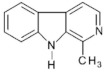	Norharman 9*H*-pyrido[4,3-b]índole	C_11_H_8_N_2_ 168.2	Soft Cheese, Beef, Mutton, Lamb Camel, Bacon, Pork Loin, Chicken, Meatball, Meat-based Infant Foods, Coffee	[16,65,68,76,86,88,92,93]
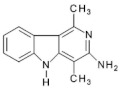	Trp-P-1 3-Amino-1,4-dimethyl-5*H*-pyrido[4,3-b]indole	C_13_H_13_N_3_ 211.3	Cheese, Meat-based Infant Foods	[87,92]
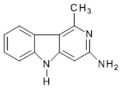	Trp-P-2 3-Amino-1-methyl-5*H*-pyrido[4,3-b]indole	C_12_H_11_N_3_ 197.2	Cheese, Meat-based Infant Foods	[87,92]
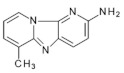	Glu-P-1 2-Amino-6-methyldipyrido[1,2-a:3′2′-*d*]imidazole	C_11_H_10_N_4_ 198.3	White Wine, Red Wine	[79]
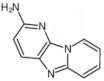	Glu-P-2 2-Amino-dipyrido[1,2-a:*3′2′*-*d*]imidazole	C_10_H_8_N_4_ 184.3	Red Wine	[79]
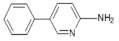	Phe-P-1 2-Amino-5-phenylpyridine	C_11_H_10_N_2_ 170.2	Beef	[94]

**Table 2:** Heterocyclic amine inhibitory effect of some plants.

Plant (Part)	Scientific Name	Extract/Dose Level	Product	Process	Targeted HAAs	Types of Inhibitors	Inhibition	Reference
Black pepper (Seed)	*Piper nigrum*	Powder 1%	Tilapia Fillets	Fried at 180 °C for 8 min	PhIP, MelQx	Antioxidants	PhIP 100% MelQx 100%	[146]
Salt cedar (bark)	*Tamarix ramosissima*	60% ethanol 0.45 mg/g	Lamb Patties	Grilled in oven at 200 °C for 20 min	PhIP	Flavonoids	PhIP 72.92%	[147]
Nutmeg (Seed)	*Myristica fragrans*	Essential oil 0.04%	Beef Patties	Grilled for 5 min at 75 °C internal temp.	MeIQ	Antioxidants	MeIQ 100%	[148]
Sowthistle (Whole)	*Sonchus olearleu*	Powder 0.5%	Pork Patties	Pan-fried at 230 °C for 105 min	IQ, Harman, Norharman	Antioxidant and total phenolic content	IQ 39% Harman 67% Norharman 63%	[149]
yellow gentian (Leaf)	*gentiana lutea*	Methanol 2%	Meat	Thermally processed	IQ, PhIP	Antioxidants	IQ 72% PhIP 76%	[150]
yellow gentian (Root)	*gentiana lutea*	Methanol 2%	Meat	Thermally processed	IQ, PhIP	Antioxidants	IQ 58% PhIP 80%	[150]
Florist’s daisy (Flower)	*Chrysanthemum morifolium*	70% Ethanol 0.2%	Goat Meat Patties	Deep fat fried at 225 °C	PhlP, Norharman, Harman, MelQx	Quercetin glucoside, Kaempferol, Paeoniflorin, 3-Caffeoylquinic acid, and Cyanidin 3-O-galactoside	PhlP 62% Norharman 59% Harman 58% MelQx 52%	[151]
Turmeric (Root)	*Curcuma longa*	80% Ethanol 3%	Beef Cubes	Electrically grilled at 240 °C for 10 min	IQ, IQx, 7,8 DiMeIQx, PhIP, Harman, Norharman, AαC	Curcumin, Desmethoxycurcumin, Bisdesmethoxycurcumin	Total HAAs 75.4%	[33]
Sichuan pepper (Seed)	*Zanthoxylum bungeanum*	Powder 1%	Grilled Beef Patties	Powder mixed with beef. Patties grilled at 225 °C for 10 min.	PhlP, IQx, MelQx, 4,8 Di MelQx,	Phenolic compounds	PhlP 90% IQx 100% MelQx 81% 4,8Di MelQx 89%	[152]
Hawthorn (Fruit, Flower, Leaves)	*C. pinnatifida*	Aqueous Extract Chicken: 1% Beef: 0.5%	Chicken Breasts and Beef	Pan cooking or oven cooking at 150, 200, and 250 °C	IQx, IQ, MelQx MelQ, 4,8 DiMelQx, 7,8 DiMelQx, PhlP, Harman, Nor-Harman, TrP2	Flavonoids Proanthocyanins	Total HAAs Chicken: 19–97% Beef: 42–100%	[140]
Artichoke (Flower buds)	*Cynara scolymus*	Commercial Extract 1%	Beef and Chicken Breast	Meat was cooked at 150, 200, and 250 °C	IQx, IQ, MelQx, AαC MelQ, 4,8 DiMelQx, 7,8 DiMelQx, PhlP, Harman, TrP2, Meαc Nor-Harman,	Mono- and di-caffeoylquinic acids, flavonoids	Total HAAs Beef: 25–98% Chicken: 14-95%	[17]
Clove (Leaves)	*Syzgium aromaticum*	Powder 0.2%	Barbecued Sucuk	Wire barbecue on charcoal	IQ, MeIQ, MeIQx	Antioxidants	IQ 41.84% MeIQx 53.84%	[76]
Chili pepper (Fruit)	*Capsicum annuum*	Powder 0.5%	Roast Beef Patties	Heated at 225 °C for 10 min on each side	PhIP, IQx, MeIQx, 4,8-DiMeIQx	Pro-oxidative, Capsaicin, polyphenolic compounds	68% PhIP Total HAAs 46%	[153]
Cinnamon (Leaves)	*Cinnamomum zeylanicum*	Powder 0.5%	Barbecued Sucuk	Wire barbecue on charcoal	IQ, IQx, MeIQ, MeIQx	Antioxidants	IQ 69.50% IQx 25% MeIQx 53.84%	[76]
Apple (Peel)	*Malus pumila*	Polyphenol-rich Extract 0.3%	Beef Patties	Extract applied on surface at ambient conditions for 30 min prior to Frying at 223 °C for 10 time.	PhIP, MeIQx, 4,8-DiMeIQx	Proanthocyanidins	83% PhIp 68% MeIQx 56% 4,8-DiMeIQx	[115]
Bamboo (Leaves)	*Bambusoideae*	Antioxidant Mixture 2.5 mg/mL	Chemical Model System	2.08 mg of creatinine and 3.2 mg of phenylalanine, weighed and mixed in 2 mL of diethylene glycol	PhIP	Orientin, homoorientin, vitexin, and isovitex	PhIP > 50%	[154]
Pomegranate (Seed)	*Punica granatum*	Commercial Extract 0.5%	M	Charcoal barbeque	PhIP, IQ, MeIQx	Phenolic content and antioxidant capacity	68% PhIP 45% IQ 57%MeIQx	[35]
Pomegranate (Seed)	*Punica granatum*	Commercial Extract 0.5%	Chicken meatballs	Oven roasting, charcoal barbeque, and deep fat frying	PhIP, IQ, MeIQx, norharman	Phenolic content and antioxidant capacity	75% PhIP 46% IQ 49% MeIQx57% Nonharman	[35]
Grape (Seed)	*Vitis vinifera L*	Aqueous Extract 0.6%	Beef Patties	Marinated patties dispersed in sunflower oil and fried at 230 °C	MelQx, PhlP	Procyanidins polyphenols	70% MelQx 90% PhlP	[126]
Savory (leaves)	*Satureia hortensis*	Ethanol / Water (70/30 *v*/*v*) 0.5%	Beef Meat	Spice extract applied and cooked meat at 200 °C for 20 min in diethylene glycol	PhlP	Phenolic compounds	PhIP 37.31 %	[39]
Oregano (leaves)	*Origanum vulgare*	Ethanol / Water (70/30 *v*/*v*) 0.2%	Beef Meat	Spice extract applied and cooked meat at 200 °C for 20 min in diethylene glycol	PhlP	Phenolic compounds	PhIP 43.28 %	[39]
Black pepper (Seed)	*Piper nigrum*	Powder 1%	Meatball	Black pepper spread on the surface of meat for 12 h prior to frying at 225 °C	PhIP, MeIQ, 4,8-DiMeIQx	Antioxidants	100% PhIP Total HAAs 12-100%	[55]
Rosemary (Leaves)	*Rosmarinus officinalis*	20% Ethanol 0.05%–0.50%	Beef Patties	Extract added to ground beef. Cooking at 191 °C for 5 min.	MelQx, PhlP	Rosmarinic acid, carnosol, carnosic acid	91.7% MelQx 85.3% PhlP	[155]
Hibiscus	*Hibiscus sabdariffa*	Commercial Extract 0.8%	Beef Patties	Sunflower-oil-coated patties fried at 230 °C	MeIQx	Flavonoids, anthocyanins	50% MeIQx	[137]
Green Tea (Leaves)	*Camellia sinensis*	Infusion with Hot Water 10 μL of Standard	Pan-Fried Beef	6 h marinated beef grilled at 180-200 °C for 4 min on each side.	PhlP, AαC	Catechins	74% PhlP 85% AαC	[156]
Apple (Fruit)	*Malus pumila*	Ethanol–Water extract 0.1%	Beef Patties	Fried at 210 °C for 6 min on each side.	PhIP, MeIQx, 4,8-DiMeIQx	Proanthocyanidins, phloridzin, and chlorogenic acid	69% PhIp 59% MeIQx 64% 4,8-DiMeIQx	[51]
Elderberry	*Sambucus nigra*	Commercial Extract 0.1%	Beef Patties	Fried at 210 °C for 6 min on each side.	PhIP, MeIQx, 4,8-DiMeIQx	Phenolic contents galic acid	45% PhIp Total HAAs 35%	[157]
Pine (bark extract)	*Pinus maritima.*	Pycnogenol Powder 1%	Cooked Beef	Cooked at 200 °C for 20 min	MeIQx, PhIP, Norharman	Thocyanidins, catechins, anthoxanthins, epicatechins	77% MeIQx 54% PhIP 27% Nonharman	[127]
Olive oil (Fruit)	*Olea europaea*	Frying Oil	Beef Burger	Fried in fresh virgin olive oil at 200 °C for 5 min per side	PhIP, Harman, Norharman	3,4-DHPEA-EDA, 3,4-DHPEA-EA, p-HPEA-EA, p-HPEA-EDA,	Total HAAs 40–60%	[158]
Tomato (Fruit)	*Solanum lycopersicum*	Carotenoid Extract 1000 ppm	Bovine Meat Juice	Freeze dried meat juice mixed with water (1:2 ratio)	MeIQx, 4,8-DiMeIQx	β-carotene, lycopene	13% MeIQx 5% 4,8-DiMeIQx	[159]
Olive oil (Fruit)	*Olea europaea*	Virgin Olive Oil 500 mg in 2.5 mL solution	Chemical Model System	Creatinine, glycine, and glucose (1:1:0.5 ration) dissolved in Milli-Q water	IQx, MeIQx, DiMeIQx by 45, 50, and 59%	Phenolic compounds Dihydroxyphenylethanol derivatives	45% IQx 50% MeIQx 59% DiMeIQx	[160]

## Data Availability

Will be available on request from the corresponding authors.
